# When teamwork is not enough: how organizational learning and continuous improvement strengthen the teamwork–handoff relationship in hospitals

**DOI:** 10.1093/intqhc/mzag167

**Published:** 2026-10-14

**Authors:** Seung-Hwan Kim

**Affiliations:** School of Business, Embry-Riddle Aeronautical University, United States

**Keywords:** hospital handoffs, teamwork, organizational learning, continuous improvement, patient safety, quality improvement

## Abstract

**Background:**

Hospital handoffs, including shift changes and transfers across units, are vulnerable points in care delivery where communication failures may lead to information loss and preventable adverse events. Although teamwork is a well‑established driver of handoffs, prior research suggests that its effectiveness varies across organizational contexts. Organizational learning–continuous improvement (OL-CI)—defined by routine review of work processes, testing of changes, and prevention of recurring problems—may strengthen the degree to which teamwork translates into more reliable handoff processes. This study examines whether OL-CI moderates the relationship between teamwork and handoffs in a hospital.

**Methods:**

A cross‑sectional dataset of 394 hospital‑unit observations was analyzed using validated composites from the Hospital Survey on Patient Safety Culture Version 2.0 (HSOPS 2.0). Handoffs and Information Exchange served as the dependent variable, Teamwork as the independent variable, and OL-CI as the moderator. All focal variables were mean‑centered and entered into a moderation model using the PROCESS macro (Model 1). Conditional effects were evaluated at low, median, and high levels of OL-CI.

**Results:**

The model accounted for 50.7% of the variance in handoff quality. Teamwork demonstrated a significant positive association with handoffs (*b* = 0.416, *P* < .001), and OL-CI independently predicted better handoffs (*b* = 0.458, *P* < .001). The Teamwork × OL-CI interaction was significant (*b* = 0.223, *P* = .004), indicating that OL-CI strengthened the effect of teamwork on the quality of handoffs. Simple‑slope analyses showed a progressively stronger relationship between teamwork and handoffs at low (*b* = 0.372), median (*b* = 0.423), and high (*b* = 0.458) OL-CI levels, demonstrating that teamwork yields greater gains in units with stronger OL-CI practices.

**Conclusion:**

The findings indicate that teamwork contributes to higher-quality handoffs, with greater benefits in organizations that support continuous learning and improvement. To promote reliable handoffs, hospitals should combine teamwork development and structured handoff tools with established practices for process review, recurring feedback, and the prevention of repeated communication failures. Longitudinal research is recommended to clarify these relationships and determine which organizational practices are most effective in sustaining high-quality handoffs over time.

## Introduction

Hospital handoffs are vulnerable points in clinical care because they require accurate and efficient transfer of patient information and professional responsibility. Handoffs occur across operational boundaries, particularly during intra-unit shift changes and inter-unit patient transfers. Although clinicians and workflows differ across departments, both types of transitions create risks of information loss, care delays, fragmented coordination, and preventable adverse events. Recent research suggests that hospital handoffs should address both team processes and broader organizational factors that safeguard information transfer [1, 2].

Teamwork can improve handoff reliability by helping healthcare professionals coordinate tasks, support colleagues, and maintain respectful working relationships. Recent hospital handoff frameworks such as SBAR [3] and I-PASS [4] provide the structural and standardized blueprint for clinical communication during handoff processes. Within these frameworks, teamwork serves as the primary engine of hospital handoffs, though its effectiveness remains bound to broader systemic inputs such as leadership engagement, institutional climates of learning, and technological infrastructure [1]. Bordin-Wosk *et al.* [5] found persistent differences in handoff performance among teams receiving similar training or using the same standardized tools. This variability suggests that handoff frameworks and team training may not be sufficient to ensure consistently reliable handoffs.

Organizational learning–continuous improvement (OL-CI) may help explain this variability. In the Hospital Survey on Patient Safety Culture Version 2.0 (HSOPS 2.0), OL-CI indicates the extent to which hospital units learn from problems, implement changes, evaluate their effectiveness, and prevent recurring safety concerns [6, 7]. These practices may help units identify weaknesses in handoffs, refine information-exchange routines, and sustain effective teamwork. Brady *et al.* [8] similarly emphasize feedback mechanisms and interprofessional engagement in maintaining safer hospital environments. Other evidence indicates that OL-CI infrastructures can increase safety-event reporting and professionals’ willingness to learn from errors [9]. Systematic reviews also identify teamwork and learning as recurring strengths of patient safety culture [10, 11].

Despite its conceptual relevance, limited evidence exists on whether OL-CI influences the relationship between teamwork and hospital handoffs. This study addresses that gap by examining whether OL-CI moderates the association between Teamwork and Handoffs and Information Exchange, using HSOPS 2.0 composite scores. “Handoffs” refers to the broader HSOPS 2.0 Handoffs and Information Exchange composite, which includes information exchange during intra-unit shift changes and inter-unit patient transfers. We test whether the positive relationship between teamwork and handoffs is stronger in units with higher OL-CI.

### Handoffs and information exchange

Handoffs are critical to patient safety because they transfer both clinical information and responsibility for care. They frequently occur under time pressure, interruptions, competing demands, and uncertainty about patients’ conditions. Differences in professional roles and perspectives can further complicate the exchange. When changes in clinical status, pending test results, medications, or care priorities are not communicated clearly, treatment may be delayed and patients may experience preventable harm [1, 5, 12, 13].

Hospitals have adopted structured frameworks to reduce variation in information exchange. SBAR organizes communication around the patient’s situation, background, clinical assessment, and recommended action [3]. I-PASS similarly emphasizes illness severity, a patient summary, pending actions, contingency planning, and synthesis by the receiving clinician [4]. These frameworks can reduce communication failures and medical errors [4, 14, 15], but their effectiveness depends on implementation. Training, coaching, leadership support, and ongoing monitoring are generally necessary to integrate a framework into routine practice [4]. Standardized frameworks may have limited effects when team and organizational conditions do not support consistent use.

This study measures handoffs through the HSOPS 2.0 Handoffs and Information Exchange composite, which addresses information exchange during shift changes and transfers across units [6, 7]. Therefore, the analysis concerns hospital handoffs broadly and should not be interpreted as applying exclusively to either shift changes or inter-unit transfers.

### Teamwork and handoffs and information exchange

Teamwork concerns how staff coordinate their work, assist colleagues, and interact respectfully. Theoretical models identify communication, coordination, leadership, and mutual performance monitoring as important determinants of handoff accuracy [1, 4]. The HSOPS 2.0 Teamwork composite measures these behaviors among staff within the same hospital unit [6, 7].

These behaviors are important because responsibility cannot be transferred safely through one-way information delivery. Clinicians must ask questions, clarify uncertainty, confirm priorities, and coordinate subsequent actions. Handoffs are therefore team activities rather than simple exchanges of clinical facts. Analyses of safety-event reports indicate that communication and teamwork problems account for many failures during transfers from emergency departments to inpatient units. Common problems include interruptions, unstructured exchanges, and differences in clinicians’ understanding of patients’ conditions and care needs [13].

Structured frameworks such as SBAR [3] and I-PASS [4] reduce variability, but their effectiveness depends on teamwork. Evidence suggests that I-PASS can reduce errors, although implementation commonly requires team training, coaching, and shared learning [2]. Structured tools provide the greatest benefit when accompanied by strategies such as role clarity and leadership engagement [2].

Communication and teamwork are related but distinct in HSOPS 2.0. Communication Openness and Communication About Error are separate patient safety culture dimensions [6, 7]. Communication Openness concerns whether staff can speak up and raise safety concerns, whereas Communication About Error addresses whether staff receive information about errors and discuss prevention. Both concepts closely tie to handoffs and overlap with Teamwork and OL-CI. Rather than including every relevant HSOPS 2.0 composite, we focused this study strictly on Teamwork and Handoffs and Information Exchange. We then ran sensitivity analyses (detailed in sections Materials and Methods and Results) to verify that our findings remain robust when accounting for these two communication composites.

### OL-CI and handoffs and information exchange

Hospital units differ in how they respond to safety problems. Some regularly review problems, introduce changes, assess whether those changes work, and take steps to prevent recurrence. Others may address individual events without identifying broader patterns or making lasting changes. In HSOPS 2.0, these practices are captured by the OL-CI composite [6, 7].

OL-CI may improve handoffs in several ways. Reviewing safety concerns can identify recurring information gaps, delays, or misunderstandings during shift changes and patient transfers. Units can then revise handoff procedures and determine whether the changes fit local workflows. Collective event review may also reveal patterns that are difficult to identify from a single failure, while feedback can reinforce effective practices and reduce recurring communication problems [8, 9, 15].

Research supports the value of event-reporting systems, feedback processes, and learning networks in helping hospitals learn from safety events [8, 9]. Such mechanisms enable concerns raised by individual staff members to inform unit-level practice. In this way, OL-CI may help units move beyond isolated responses and develop more reliable information-exchange processes.

### The moderating role of OL-CI

Teamwork may be associated with better handoffs, but its benefits are unlikely to be the same in every hospital unit. Staff may work well together during individual handoffs, yet recurring problems may persist if the unit does not review failures, test possible solutions, or assess whether changes have been effective. OL-CI may shape the extent to which effective teamwork produces lasting improvements in the quality of handoffs.

Teamwork can support the immediate exchange of information and coordination of care, whereas OL-CI can enable units to evaluate and improve that work over time. For example, staff may notice that important information is repeatedly omitted during shift changes. In a unit with strong OL-CI, they are more likely to investigate the causes, revise the process, and monitor results. Under weaker learning conditions, the problem may be repeatedly recognized without producing sustained change.

Structured handoff frameworks illustrate this potential moderation. Teamwork supports their coordinated use, but their effects may be greater in units that routinely evaluate performance and adapt practices. Training, audit and feedback, and local adaptation help teams identify breakdowns, refine routines, and sustain improvements [4, 8, 15]. Event reviews and cross-unit improvement activities may further strengthen teamwork by aligning expectations and reducing recurring communication problems during transfers [8, 9]. Teamwork may improve individual handoffs, while OL-CI helps units refine and sustain effective practices. We therefore expect teamwork to be positively associated with handoffs and this association to be stronger at higher levels of OL-CI.

## Materials and methods

### Study design and setting

This cross-sectional observational study used secondary data to examine whether OL-CI moderates the relationship between Teamwork and Handoffs and Information Exchange. All primary variables were measured using validated HSOPS 2.0 composites, which provide an established framework for examining patient safety culture [6, 7]. Analyses were conducted in SPSS using the PROCESS macro, Model 1. The model tested a first-order interaction within a linear regression framework. Predictors were mean-centered to improve interpretation of the coefficients and interaction term.

### Participants and sample

The analytical sample included 394 unit-level observations with complete data on the focal HSOPS 2.0 composites. Observations missing any focal variable were excluded through listwise deletion. The PROCESS output confirmed a final sample of 394 after predictor centering and creation of the interaction term.

### Measures

#### Handoffs and information exchange (dependent variable)

The dependent variable was the three-item HSOPS 2.0 Handoffs and Information Exchange composite. Two items address information exchange and sufficient time during shift changes, while one addresses the transfer of important patient-care information across hospital units. The composite therefore represents perceived information exchange during both intra-unit shift changes and inter-unit transfers. “Handoffs” is used as a shortened label for this broader construct rather than as a measure of inter-unit transfers alone.

#### Teamwork (independent variable)

Teamwork was measured with the three-item HSOPS 2.0 Teamwork composite. It captures routine coordination, backup behavior, and respectful conduct, which are foundational components of safety-relevant teamwork.

#### OL-CI (moderator)

OL-CI was measured with the three-item HSOPS 2.0 OL-CI composite. It reflects the extent to which units learn from problems, implement changes, and evaluate their effects.

#### Communication composites of HSOPS 2.0

We recognize that communication is discussed extensively in this study, but the two communication composites from HSOPS 2.0, Communication Openness and Communication About Error, are not included in the model. Sensitivity analyses added both composites as covariates to determine whether the OL-CI moderation effect remained robust after accounting for broader communication conditions. The results are reported in the Results section.

### Psychometric considerations and data preparation

The HSOPS 2.0 composites have demonstrated acceptable internal consistency and site-level reliability. Confirmatory factor analyses conducted during multisite pilot testing also support their construct validity [7]. Teamwork and OL-CI were mean-centered before the interaction term was calculated. Consistent with Hayes’ recommendations for PROCESS Model 1 [16], conditional effects were evaluated at the 16th, 50th, and 84th percentiles of the OL-CI distribution.

### Ethical considerations

The study used a de-identified secondary dataset from existing safety culture surveys. Under standard guidelines governing secondary analyses of anonymized information, the project did not meet the regulatory definition of human-subject research and posed minimal participant risk. Institutional procedures were followed to document this determination. Measurement and analytical procedures followed AHRQ HSOPS 2.0 documentation to protect confidentiality and ensure standardized score interpretation [6].

### Hypotheses development

#### Impact of teamwork

Units reporting higher levels of teamwork (effective collaboration, mutual help, respectful conduct) will report higher quality on the Handoffs and Information Exchange domain.

*H1: Teamwork is positively associated with the quality of Handoffs and Information Exchange.*

#### Impact of OL-CI

Units demonstrating higher levels of OL-CI (regular process review, evaluation of changes, and prevention of recurring safety issues) will report higher quality of handoffs.

*H2: OL-CI is positively associated with the quality of Handoffs and Information Exchange.*

#### The moderating effect of OL-CI

In units with higher OL-CI, teamwork behaviors are expected to translate into more reliable, complete, and timely handoffs.

*H3: OL-CI moderates the relationship between Teamwork and Handoffs and Information Exchange, such that the positive association between Teamwork and Handoffs and Information Exchange is stronger when OL-CI is higher.*

## Results

The moderated regression analysis demonstrated that the overall model was statistically significant, F(3, 390) = 133.731, *P <* .001, with *R*^2^ = .5071 (adjusted *R*^2^  *=* .5033), accounting for 50.71% of the total variance in perceived handoffs. Beyond the significant positive main effects of both Teamwork (unstandardized *b = *0.416, SE* = *0.066*, t = *6.34, *P* < .001, 95% CI [0.287, 0.545]) and OL-CI (unstandardized *b = *0.458, SE = 0.058, *t =* 7.96, *P <* .001, 95% CI [0.345, 0.571]), the critical Teamwork × OL-CI interaction was significant (unstandardized *b = *0.223, SE = 0.076, *t =* 2.93, *P =* .004, 95% CI [0.073, 0.372]), indicating that the positive association between Teamwork and Handoffs becomes stronger as OL-CI increases. Table 1 shows these regression results.

**Table 1:** Regression results.^a^

Variable	*b*	SE	*t*	*P*	LLCI	ULCI
**Constant**	3.639	0.009	403.221	0.000***	3.621	3.657
**Teamwork (IV)**	0.416	0.066	6.344	0.000***	0.287	0.545
**OL-CI (moderator)**	0.458	0.058	7.960	0.000***	0.345	0.571
**Teamwork × OL-CI**	0.223	0.076	2.928	0.004**	0.073	0.372

a b, unstandardized coefficient; SE, standard error; LLCI/ULCI, lower/upper bounds of the 95% confidence interval.

** P < .01.

*** P < .001.

Conditional effects of Teamwork on Handoffs and Information Exchange were probed at the 16th, 50th, and 84th percentiles of OL-CI (values of −0.200, 0.031, and 0.187 on the centered scale). At low OL-CI (−0.200), Teamwork was positively associated with handoffs (unstandardized *b = *0.372). At the median OL-CI (0.031), the association was stronger (unstandardized *b = *0.423). At high OL-CI (0.187), the association was strongest (unstandardized *b = *0.458). These results are summarized in Table 2.

**Table 2:** Conditional effects of OL-CI.^a^

OL-CI	Effect	SE	*t*	*P*	LLCI	ULCI
**Low (–0.2002)**	0.372	0.061	6.113	0.000***	0.252	0.491
**Med (0.031)**	0.423	0.067	6.350	0.000***	0.292	0.554
**High (0.1874)**	0.458	0.073	6.294	0.000***	0.315	0.601

a LLCI/ULCI, lower/upper bounds of the 95% confidence interval; SE, standard error.

*** P < .001.

Figure 1 presents the simple slope analysis of the conditional effects. The simple slope analysis demonstrates that while teamwork is positively associated with handoffs at all levels of the moderator, this relationship strengthens as OL-CI increases. The pattern of simple slopes is consistent with a positive buffering/leveraging effect of OL-CI: in units with stronger OL-CI practices, Teamwork improvements translate into larger gains in the quality of handoffs.

**Figure 1:**
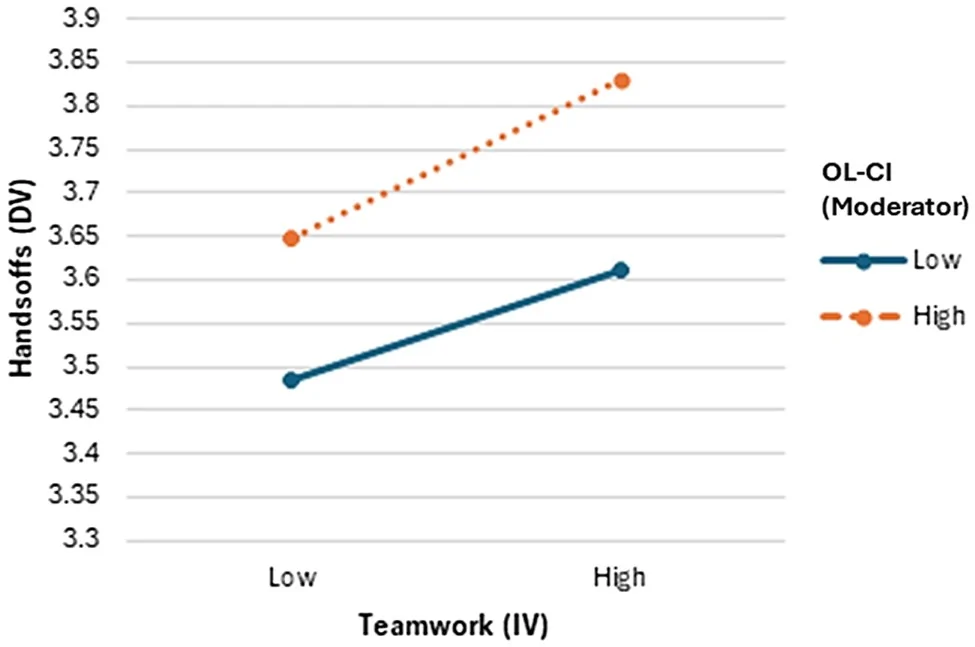
Simple slopes of the moderating effect of OL-CI.

For the sensitivity analyses, results from the model that included Communication Openness and Communication About Error showed that the Teamwork × OL-CI interaction remained significant (unstandardized *b = *0.221, SE = 0.076, *t = *2.915, *P = *.004, 95% CI [0.072, 0.370]). The composites explained an additional 1.8% of variance in Handoffs and Information Exchange. Collinearity diagnostics were within acceptable ranges, with VIF values ranging from 1.411 to 4.990. These results support the robustness of the principal finding that the positive association between Teamwork and Handoffs and Information Exchange becomes stronger as OL-CI increases.

## Discussion

### Principal findings

This study examined whether OL-CI strengthens the relationship between teamwork and handoffs in a hospital. Teamwork and OL-CI each had significant positive associations with Handoffs and Information Exchange. More importantly, OL-CI moderated the teamwork–handoffs relationship. Units with stronger OL-CI exhibited a steeper positive association, indicating that OL-CI amplifies the benefits of teamwork.

These findings suggest that teamwork alone may be insufficient to ensure reliable handoffs; instead, teamwork is most effective when embedded within a robust learning environment that reviews processes, evaluates changes, and prevents recurring communication defects.

### Limitations

One strength of the study is its use of HSOPS 2.0, which incorporates just-culture principles and improved psychometric properties. The moderation model explained a substantial proportion of variance, and the conditional effects were consistent across OL-CI levels. Nevertheless, several limitations warrant consideration.

First, the cross-sectional design precludes causal inference. The analysis cannot rule out reciprocal relationships among teamwork, OL-CI, and perceived handoff quality. Longitudinal studies are needed to establish temporal order.

Second, the primary analysis was limited to three HSOPS composites. Although sensitivity analyses controlled for Com­munication Openness and Communication About Error, patient safety culture dimensions overlap conceptually. Con­sequently, the estimated coefficients should not be interpreted as entirely independent effects.

Third, Handoffs and Information Exchange composite combines shift-change items with an item concerning transfers across hospital units. Although these items form a validated composite, the two handoff contexts involve different participants, workflows, and communication demands. The composite does not show whether the relationships differ between shift changes and inter-unit transfers. Future studies should evaluate these contexts separately with multi-item, context-specific measures.

Finally, HSOPS 2.0 uses broad unit categories to accommodate diverse hospital settings. These categories may not correspond precisely with individual hospitals’ organizational structures. Aggregating scores within broad classifications may obscure differences among units or introduce variation into unit-level estimates. However, detecting the proposed relationship despite this limitation may indicate that the findings are robust across diverse hospital structures. Research using hospital-specific unit classifications could determine whether closer organizational alignment produces stronger or more differentiated associations.

### Interpretation within the context of the wider literature

The findings align with frameworks describing handoffs as team-based activities situated within broader organizational contexts. Existing literature identifies teamwork as a proximal contributor to safe handoffs but also documents contextual variability. The observed moderation effect helps explain why structured tools such as I-PASS and SBAR may achieve different outcomes across settings. Organizational climates of learning appear not only to support handoffs directly but also to strengthen the extent to which teamwork produces reliable information exchange. National HSOPS 2.0 reports often show higher scores for Teamwork than for Handoffs and Information Exchange. The present findings offer one possible explanation: strong teamwork within units may not translate fully into effective transitions when processes for reviewing problems, providing feedback, and coordinating improvement are underdeveloped.

### Implications for policy, practice, and research

The results support a dual-track strategy for improving hospital handoffs. Healthcare leaders should continue to strengthen teamwork through training, structured communication tools, leadership support, and role clarity. These efforts should be paired with institutionalized OL-CI routines, including regular transition-of-care debriefings, cross-unit learning collaboratives, audit-and-feedback cycles, and rapid testing of process changes.

Policies supporting nonpunitive event reporting, open communication, and shared learning may further improve handoff reliability. Hospitals should also monitor whether handoffs are applied consistently, fit local workflows, and produce measurable improvements rather than treating initial implementation as sufficient.

Researchers should use longitudinal and intervention designs to examine causal pathways. Future studies could incorporate the implementation of structured frameworks such as SBAR [3] and I-PASS [4] and test interventions designed specifically to strengthen OL-CI. Examining additional HSOPS 2.0 dimensions may also identify conditions that amplify or weaken the benefits of teamwork.

## Conclusion

This study provides evidence that teamwork contributes most effectively to reliable hospital handoffs when supported by OL-CI. Using HSOPS 2.0 composites, the analysis showed that OL-CI was positively associated with Handoffs and Information Exchange and amplified the positive relationship between teamwork and handoffs.

Hospitals should therefore pair teamwork development and structured handoff frameworks with institutionalized learning and improvement routines. The findings contribute to patient safety culture theory by demonstrating the importance of organizational context: team processes are more likely to achieve their potential in environments that systematically review problems, evaluate changes, and sustain improvement. Longitudinal and implementation research should identify the learning mechanisms most effective for maintaining reliable handoffs at scale.

## Data Availability

No new data were generated or analyzed in support of this review.
